# Polygenic Inheritance for Common Comorbidities Associated With Congenital Heart Disease

**DOI:** 10.1016/j.jacadv.2025.101673

**Published:** 2025-03-21

**Authors:** Jamie-Lee M. Thompson, Ingrid S. Tarr, Emma M. Rath, Michael Troup, Eddie K.K. Ip, Congenital Heart Disease Synergy Study Group, Sally L. Dunwoodie, David Winlaw, Eleni Giannoulatoudslo, Natasha Nassar, Edwin Kirk, Gavin Chapman, Gillian Blue, Gary Sholler, Samantha Lain, Gillian M. Blue, David S. Winlaw, Sally L. Dunwoodie, Eleni Giannoulatou

**Affiliations:** a Victor Chang Cardiac Research Institute, Darlinghurst, Sydney, Australia; b School of Clinical Medicine, St Vincent's Healthcare Clinical Campus, Faculty of Medicine and Health, UNSW Sydney, Kensington, Australia; c Heart Centre for Children, The Children's Hospital at Westmead, Sydney, Australia; d Sydney Medical School, The University of Sydney, Sydney, Australia; e Lurie Children's Hospital of Chicago, Chicago, Illinois, USA; f Feinberg School of Medicine, Northwestern University, Chicago, Illinois, USA

**Keywords:** comorbidities, congenital heart disease, polygenic risk score

**What is the clinical question being addressed?**

Does polygenic inheritance play a role in the development of common comorbidities in individuals with CHD?

**What is the main finding?**

Polygenic risk scores for hypertension, type 2 diabetes, and obesity were significantly higher in CHD patients compared to healthy controls.

Congenital heart disease (CHD) encompasses any structural abnormalities that are present in the cardiovascular system at birth and has an incidence rate of 1 in 100 live births. Individuals with CHD often have an added burden of comorbidities, with approximately two-thirds of patients affected by co-occurring medical conditions.^1^ Low birthweight is a commonly observed comorbidity in infants with CHD, while common adult-onset comorbidities include hypertension and metabolic disorders.^1^ The presence of one or more comorbidities can significantly impact the overall health and quality of life for individuals with CHD, requiring additional care and management. Close monitoring and early intervention are essential for managing the primary heart defect and associated comorbidities effectively. The aim of this project was to assess the role of polygenic inheritance in the development of common comorbidities by applying polygenic risk scores (PRSs) for these comorbidities to a cohort of CHD patients.

## Methods

Two separate cohorts of CHD patients that have undergone whole genome sequencing were used for this study. The first cohort comprised of 184 patients recruited at the Victor Chang Cardiac Research Institute and from the Australian Genomics Health Alliance Cardiovascular Flagship.^2^ A healthy elderly control cohort (3,823 individuals) from the Medical Genome Reference Bank was used for comparison.^3^ A second cohort of 486 CHD patients from the Genomics England database was used for validation^4^ with the 1,000 Genomes participants used for comparison (503 individuals).^5^ All subjects had European ancestry, as confirmed by principal component analysis analysis with 1,000 Genomes samples, and were selected irrespective of the presence of comorbidities. Polygenic scores for each participant were calculated with PLINK (v1.9). PRSs were calculated for 4 common comorbidities: hypertension (Polygenic Score Catalog ID: PGS001838), type 2 diabetes (PGS001818), obesity (PGS001825), and birth weight (PGS001892). PRSs were transformed into percentiles of the control PRS distribution and differences in percentile distributions were compared using a Mann-Whitney *U* test. Benjamini and Hochberg correction for multiple testing was separately applied to *P* values for each cohort.

## Results

**Figure 1:**
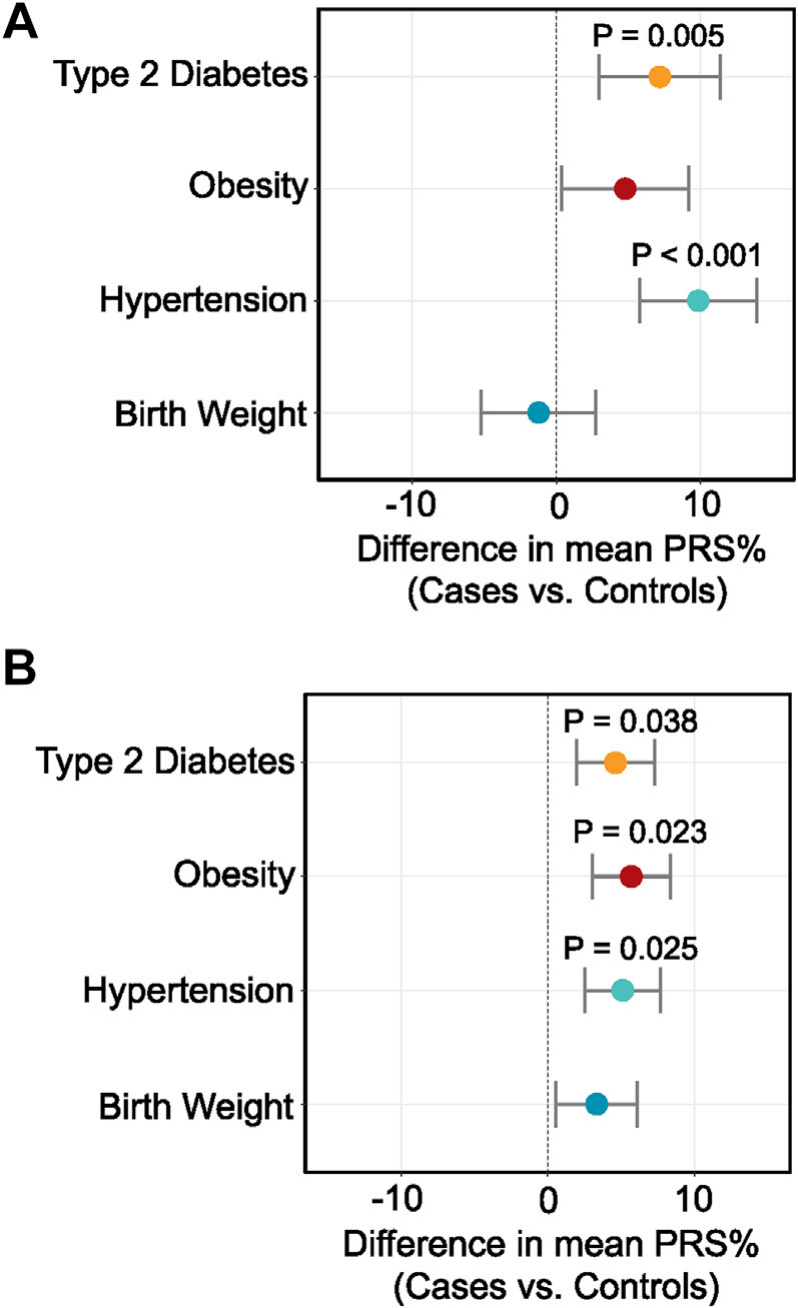
**Polygenic Risk Scores for Common Comorbidities in Patients With Congenital Heart Disease Compared With Healthy Control Subjects** (A) PRS for comorbidities in Victor Chang Cardiac Research Institute CHD patients (n = 184). (B) PRS for comorbidities in Genomics England patients (n = 486). Dots show the difference in mean PRS between cases and controls where scores have been transformed into percentiles based on the control distribution (Medical Genome Reference Bank n = 3,823 and 1000G n = 503), and lines show the 95% CI. CHD = congenital heart disease; PRS = polygenic risk score.

Results from the first cohort showed that PRS for several common CHD comorbidities including hypertension, type 2 diabetes, and obesity were higher in patients than comparable healthy controls, with significant associations for hypertension and type 2 diabetes (Figure 1A). CHD patients had a mean PRS percentile for hypertension of 59.8% (*P* < 0.001), a mean PRS percentile for type 2 diabetes of 57.2% (*P* = 0.005), and a mean PRS percentile for obesity of 54.8% (*P* = 0.083), compared to healthy controls (mean PRS percentile of 50%). The validation cohort supported this, with significant associations for PRS for hypertension (55.1% mean PRS percentile; *P* = 0.025), type 2 diabetes (54.6% mean PRS percentile; *P* = 0.038), and obesity (55.7% mean PRS percentile; *P* = 0.023) (Figure 1B). A birth weight PRS showed no significant association in either cohort. The first cohort had a mean PRS percentile of 48.8% (*P* = 0.402) and the validation cohort had a mean PRS percentile of 53.3% (*P* = 0.140).

## Discussion

The data presented here suggest polygenic inheritance may contribute to the presence of some CHD comorbidities and further, that CHD may exhibit a genetic overlap with these comorbidities. PRS may provide a more comprehensive understanding of a patient’s risk for development of comorbidities, specifically hypertension and type 2 diabetes.

We acknowledge that we were unable to perfectly match cases and controls in our discovery cohort for confounders such as sex and age due to cohort limitations. While no explicit matching was performed, we applied strict quality control measures, including genetic ancestry analysis, to minimize population structure differences. Additionally, the second cohort, which utilizes the widely adopted 1,000 Genomes data set as a control population, serves as an independent replication. This mitigates concerns that our findings could be driven by the control selection in the first cohort.

Further research is needed to validate the clinical outcomes associated with these PRSs in CHD patients, however implementing PRS in the clinical setting has the potential to improve patient outcomes and quality of life.

## Funding support and author disclosures

This work was supported by the 10.13039/501100000925National Health and Medical Research Council (NHMRC), Australia Synergy Grant to Drs Dunwoodie, Giannoulatou, Blue, and Winlaw (1181325). Dr Giannoulatou is supported by a NSW Health Early-Mid Career Cardiovascular Grant, a 10.13039/501100000925NHMRC Investigator Grant (2018360), and a Ramaciotti Health Investment Grant. Dr Dunwoodie is supported by an 10.13039/501100000925NHMRC Investigator Grant (2007896) and a NSW Health Cardiovascular Senior Scientist Grant. Dr Blue is supported by a NSW CVRN Career Advancement Grant. Genomic data for this study were accessed through Australian Genomics, funded by the 10.13039/501100000925NHMRC (1113531, 2000001) and the MRFF, administered by the Murdoch Children's Research Institute. All other Australian Genomics Cardiovascular Disorders Flagship was funded by the MRFF Genomic Health Futures Mission (EPCD000028). The authors have reported that they have no relationships relevant to the contents of this paper to disclose.
